# Medial plantar artery perforator and anterolateral thigh perforator flaps for complex forefoot reconstruction: A retrospective case series

**DOI:** 10.1016/j.jpra.2026.03.038

**Published:** 2026-04-03

**Authors:** Yue-liang Zhu, Xiao-qing He, Feng-yi Jiang, Yuan-jie Li, Wen-long He, De-hong Zhang, Kai-xuan Dong

**Affiliations:** a Department of Orthopedic Surgery, The Second Affiliated Hospital, Zhejiang University School of Medicine, Orthopedics Research Institute of Zhejiang University, Clinical Research Center of Motor System Disease of Zhejiang Province, Zhejiang Key Laboratory of Motor System Disease Precision Research and Therapy, State Key Laboratory of Transvascular Implantation Devices, Hangzhou, Zhejiang 310009, China; b Department of Orthopedics, 920th Hospital of the Joint Logistic Support Force, People’s Liberation Army of China, Kunming, Yunnan 650032, China; c Department of Orthopedics, The First People’s Hospital of Yunnan Province, The Affiliated Hospital of Kunming University of Science and Technology, The Key Laboratory of Digital Orthopedics of Yunnan Province, The Clinical Medicine Center of Spinal and Spinal Cord Disorders of Yunnan Province, Kunming, Yunnan 650032, China; d Department of Orthopedics, Sino-German Orthopedic Hospital of Yunnan Province, Kunming, Yunnan 650032, China

**Keywords:** Medial plantar artery perforator flap, Anterolateral thigh perforator flap, Forefoot, Defect repair, Microsurgical technique

## Abstract

**Background:**

Soft-tissue coverage of the forefoot, especially for irregular and extensive skin defects, is challenging for reconstructive surgeons because of the complex biomechanical properties and the high sensitivity of the plantar region. In this study, the efficacy of a combined approach utilizing a medial plantar artery perforator flap (MPAPF) and an anterolateral thigh perforator flap (ALTPF) for complex forefoot reconstruction was evaluated.

**Methods:**

A retrospective case series was conducted. From May 2018 to October 2023, six patients with complex forefoot defects underwent reconstruction using both an MPAPF and an ALTPF. The cohort comprised four males and two females aged 19–67 years. Data on patient demographics, injury etiology, flap size and survival, time to initial weight-bearing, and restoration of protective sensation were analyzed using descriptive statistics.

**Results:**

All the flaps were free transplants, and no cases of vascular crisis were reported. MPAPF sizes ranged from 4.0 cm × 6.5 cm to 8.0 cm × 11.5 cm, whereas ALTPF sizes varied from 6.5 cm × 8.5 cm to 11.5 cm × 15.0 cm. During 8–27 months of follow-up, all patients achieved full weight-bearing within 3 months (mean 11.2 weeks). Protective sensation was restored in 83.3% (5/6) of the patients within 6 months and in the remaining 16.7% (1/6) of the patients within 8 months. The flaps exhibited good color and texture, and durable, sensitive coverage was observed in all the cases, with no recurrence of ulceration or major complications.

**Conclusion:**

The combined use of an MPAPF and ALTPF provides a feasible and promising strategy for complex forefoot reconstruction, resulting in reliable functional and sensory recovery with satisfactory aesthetic outcomes.

## Introduction

The reconstruction of soft-tissue defects in the forefoot remains a formidable challenge in reconstructive surgery. This region is endowed with unique biomechanical properties, featuring thick, glabrous skin, shock-absorbing fibrofatty tissue, and dense fibrous septa that anchor the skin to deep structures and are essential for maintaining weight-bearing functionality and gait mechanics.^1^ Consequently, the loss of this specialized tissue due to trauma, infection, or chronic pathologies, including diabetic complications, often exposes vital structures and compromises the weight-bearing function of the foot, necessitating a reconstruction for both anatomical restoration and functional preservation.

Conventional methods for forefoot reconstruction include skin grafting, local pedicled flaps, and free flap transplantation.^2^, ^3^, ^4^, ^5^, ^6^, ^7^ Although these methods are widely used in clinical practice, they have several limitations. Skin grafts lack the necessary durability and cushioning and often succumb to breakdown under mechanical stress. Local pedicled flaps may provide limited coverage, while many common free flaps, such as latissimus dorsi, often result in a bloated contour and fail to restore protective sensation, making patients vulnerable to recurrent ulceration. These problems can affect the load-bearing and walking functions of the foot and impact its appearance. Consequently, developing more effective and durable reconstruction methods that address both functional and aesthetic requirements represents a critical focus in contemporary reconstructive surgery research and practice, particularly for plantar weight-bearing surface injuries.

The medial plantar flap, initially described by Harrison and Morgan in 1981 as the ‘instep island flap’ for resurfacing plantar defects, laid the foundation for subsequent refinements in plantar reconstruction using this specialized tissue.^8^ In 2007, Koshima et al. described the use of the medial plantar artery perforator flap (MPAPF).^9^ Since its introduction, the MPAPF has been considered an ideal option for reconstructing soft-tissue defects of the fingers and feet because of its proper thickness, similar anatomical structure, sufficient flap size, and low donor-site morbidity.^10^, ^11^, ^12^, ^13^ However, the application of the MPAPF for complex, extensive forefoot defects is less frequently reported. A significant limitation is its relatively small size and limited arc of rotation, which often precludes its use as a solitary flap for larger, irregular wounds. This aligns with the findings of Scaglioni et al., who emphasized the need for alternative strategies when substantial forefoot tissue loss is addressed.^14^ To overcome this constraint, a combination of flaps may be needed. Therefore, we used an MPAPF connected with an anterolateral thigh perforator flap (ALTPF) to repair forefoot soft-tissue defects, a combined technique approach that has yielded promising clinical outcomes.

This study aimed to describe the surgical technique, assess the feasibility of this approach, and evaluate the preliminary functional and sensory outcomes.

## Patients and methods

This study was designed as a retrospective case series analysis of a consecutive cohort of patients who underwent complex forefoot reconstruction using a combined MPAPF and ALTPF technique between May 2018 and October 2023. This study was approved by the institutional ethics committee, and written informed consent was obtained from all participants.

The inclusion criteria were as follows: (1) presence of a complex forefoot and/or midfoot soft-tissue defect that could not be closed primarily after debridement or excision; (2) defect size or geometry precluding reconstruction with a single local or free flap; and (3) successful limb salvage, which was deemed feasible and appropriate by a multidisciplinary team.

The exclusion criteria included the following: (1) patients with severe peripheral vascular disease unsuitable for microvascular anastomosis; (2) medical comorbidities contraindicating prolonged surgery; (3) isolated hindfoot or heel defects; (4) patients lost to follow-up before the 6-month assessment; and (5) severe injuries necessitating amputation.

In accordance with these criteria, a total of six patients (4 males, 2 females; aged 39.7 ± 12.3 years) were included in the final analysis.

After admission, patients in the first stage underwent debridement and vacuum sealing drainage (VSD), and in patients with fractures, the fractures were simultaneously fixed with a Kirschner wire. The second stage involved flap transfer.

## Surgical technique

Recipient site preparation: Preoperative computed tomography angiography was performed to identify suitable perforator vessels and recipient vessels. Following complete wound debridement, the extent of the soft-tissue defect in the foot was carefully assessed. Precise measurements were taken of both the defect dimensions and the weight-bearing forefoot area to be covered by the MPAPF. Surgical dissection was then performed to isolate and expose the recipient vessels in the posterior tibial, dorsalis pedis, or anterior tibial vascular systems, ensuring adequate length and quality for microvascular anastomosis.

Flap harvest: The MPAPF was designed on the contralateral medial plantar region, matching the size and shape of the recipient defect while remaining within nonweight-bearing zones. For larger defects, the flap was extended toward the medial dorsal foot. The skin, subcutaneous tissue, and plantar aponeurosis were incised at the distal margin of the flap. The medial plantar artery perforators, accompanying veins, and cutaneous nerve branches were identified between the abductor hallucis and flexor digitorum brevis muscles beneath the aponeurosis and preserved within the flap, leaving the main nerve trunk intact. Subfascial dissection proceeded from distal to proximal between the muscle layer and the neurovascular bundle. Upon reaching the anteroinferior medial malleolus, the abductor hallucis muscle was divided. The neurovascular bundle was freed up to the junction of the proximal end of the medial plantar artery and the posterior tibial artery. The medial plantar artery was ligated and divided, and the cutaneous nerve branches were transected while preserving the continuity of the posterior tibial artery and the lateral plantar artery. If the vein diameter was small, the dissection was extended to the posterior tibial vein. The severed abductor hallucis muscle was sutured, and the donor site wound was closed with a full-thickness skin graft and secured under a bolstered dressing.

The ALTPF was harvested using our standard technique, which was consistent with previously published methods.^15^, ^16^, ^17^ Primary closure of the donor site was achieved in four patients, while two patients required skin grafting.

Wound repair: The MPAPF was inserted to resurface the critical weight-bearing forefoot, whereas the ALTPF was used to cover the adjacent plantar, arch, or dorsal defects. Under microscopic guidance, the recipient vessels were meticulously prepared and anastomosed. In all the patients, microvascular anastomosis was performed with one artery and two veins by the end-to-end technique. The nerve of the medial plantar flap was anastomosed with a branch of the posterior tibial nerve, whereas the nerve of the anterolateral thigh flap was anastomosed with a branch of the superficial peroneal nerve.

## Postoperative management and outcome assessment

All patients received standardized postoperative routine antibiotic therapy. Meticulous nursing care was implemented to prevent compression of the flaps and vascular pedicles. Early surgical exploration was performed promptly if a vascular crisis occurred.

The affected limb was routinely immobilized for 1–2 weeks postoperatively to prevent traction and spasm of the vascular pedicles, which were not weight-bearing for 4 weeks, followed by gradual partial weight-bearing progression to full weight-bearing by 3 months postoperatively. Intraoperative records included documentation of plantar defect characteristics, flap type, and dimensions. The flap survival status and complications were monitored postoperatively. During follow-up, evaluations included flap contour and texture, donor- and recipient-site complications, time to weight-bearing ambulation, and sensory recovery (assessed using the British Medical Research Council scale).

### Statistical analysis

Given the small sample size (*n* = 6) and the descriptive nature of this case series, formal inferential statistical analysis was not performed. The data are presented using descriptive statistics. Categorical variables (e.g., sex and complication rates) are expressed as counts and percentages. Continuous variables (e.g., age and follow-up period) are summarized using the range and, where appropriate, mean and standard deviation or median and interquartile range to provide a clear representation of the cohort’s characteristics and outcomes.

## Results

All the defects resulted from traumatic injuries, and the anatomical distribution of the defects included the forefoot (*n* = 2), soles and arches (*n* = 3), and forefoot with the middle sole (*n* = 1) (Table 1). All flaps were successfully transferred as free tissue transplants, with a 100% survival rate, and no major postoperative complications, such as deep infection, vascular crisis or significant hematoma, occurred. The sizes of the reconstructed flaps were as follows: MPAPFs, 4.0 cm × 6.5 cm to 8.0 cm × 11.5 cm; and ALTPFs, 6.5 cm × 8.5 cm to 11.5 cm × 15.0 cm. The combination of these two flaps allowed for the complete and tension-free closure of all extensive and irregular wounds in a single stage. During the follow-up period (range: 8–27 months), all patients achieved a return to full weight-bearing ambulation. The mean time to full weight-bearing was 11.2 weeks (range: 9–12 weeks). Sensory recovery was achieved in all patients. Protective sensation (S2) was restored in 83.3% (5/6) of the patients within 6 months postoperatively, with the remaining 16.7% (1/6) achieving sensory recovery by 8 months. Among them, the ALTPF was thinned in 3 cases because of overstaffing. The flaps demonstrated excellent tissue match in terms of color and texture, providing durable, sensitive coverage in all cases without ulcer recurrence or major complications.

**Table 1:** Patient demographics (*n* = 6).

Cases	Gender	Age	Defect site	Cause of injury	ALTPF size (cm^2^)	MPAPF size (cm^2^)	Flap types	Flap survival	Sensation	Follow-Up (months)	Complication
1	Male	25	Forefoot	Heavy objects	11×15	8.0 × 11.5	Free	Yes	Protective	25	No
2	Male	57	Soles and arches	Motor vehicle accident	9.0 × 11	4.0 × 6.5	Free	Yes	Protective	19	No
3	Female	48	Soles and arches	Motor vehicle accident	8.5 × 13	5.5 × 8	Free	Yes	Protective	27	No
4	Male	42	Forefoot with middle sole	Motor vehicle accident	8.0 × 12	6 × 8.5	Free	Yes	Protective	14	No
5	Female	37	Soles and arches	Motor vehicle accident	6.5 × 8.5	5.0 × 6.0	Free	Yes	Protective	8	No
6	Male	29	Forefoot	Bruise	9.5 × 13	7.0 × 10	Free	Yes	Protective	16	No

## Case report

**Figure 1:**
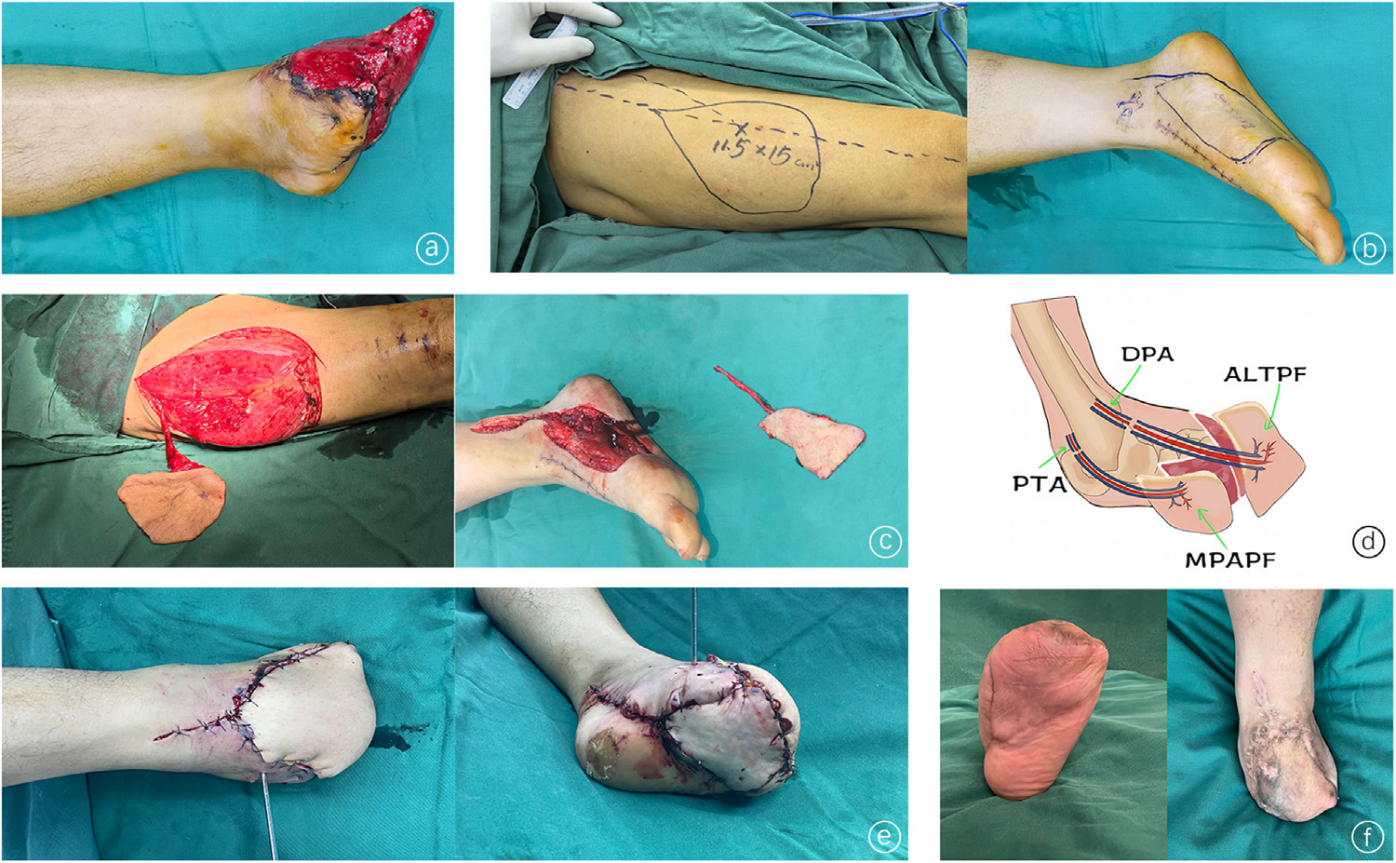
An ALTPF and an MPAPF were used to repair irregular soft-tissue defects of the forefoot. (a) Forefoot tissue defects. (b) Design of the ALTPF and MPAPF. (c) Intraoperative harvesting of the ALTPF and MPAPF. (d) Diagram of the blood vessels in the recipient area and anastomosis; the vascular pedicle of the ALTPF was anastomosed with the dorsal pedis artery (DPA) of the foot, and the vascular pedicle of the MPAPF was anastomosed with the posterior tibial artery (PTA). (e) The wound was completely repaired. (f) Follow-up revealed that the foot and flaps had a good appearance.

Case 1: A 25-year-old male patient sustained forefoot damage from heavy objects. During the first stage, emergency debridement, partial forefoot amputation, and VSD placement were performed. Two weeks later, the soft-tissue defect of the forefoot was irregular and extensive. To repair the defect, we chose a contralateral MPAPF in series with an ALTPF. The MPAPF measured 8 cm × 11.5 cm, and the ALTPF measured 11.5 cm × 15 cm; both flaps were successfully harvested. The vascular pedicle of the MPAPF was anastomosed end-to-end with the posterior tibial artery and its accompanying vein to repair the defect of the anterior plantar surface. The vascular pedicle of the ALTPF was anastomosed with the dorsal artery and its accompanying vein to repair the defect of the anterior dorsum of the foot. The wound was completely repaired, and both flaps survived. A follow-up at 25 months post-surgery revealed that the skin flaps had survived, the sensation had recovered, and no ulcers had formed (Figure 1).

**Figure 2:**
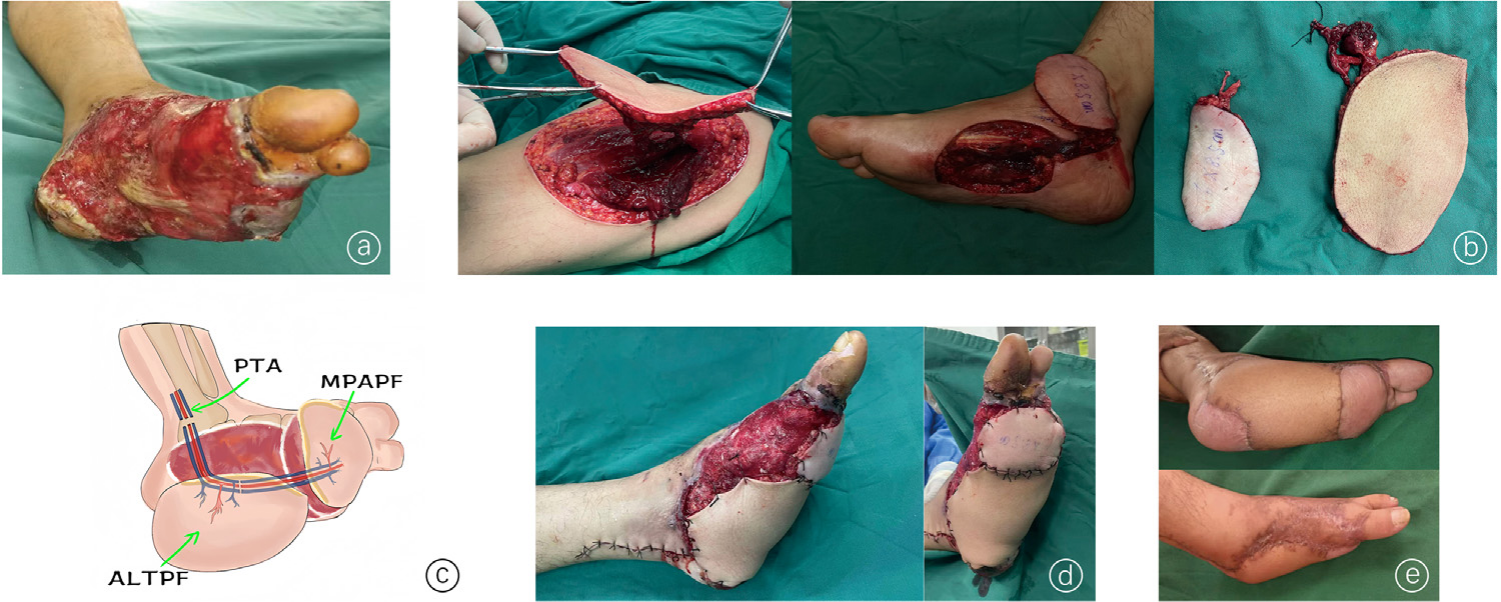
An ALTPF and an MPAPF were used to repair extensive soft-tissue defects of the soles and arches. (a) Soles and arches with soft-tissue defects. (b) Intraoperative harvesting of the ALTPF and MPAPF. (c) Diagram of the blood vessels in the recipient area and anastomosis: the ALTPF and MPAPF were connected in series using flow-through technology. The vascular pedicle of the ALTPF was anastomosed with the PTA. (d) Flaps were used to cover the main wound area. (e) Follow-up revealed that the flaps survived well, with a satisfactory appearance and no ulceration.

Case 2: A 42-year-old male patient experienced a motor vehicle accident, resulting in avulsion of the skin on his left foot and damage to the 3rd to 5th toes. Upon admission, emergency debridement, amputation of the 3rd to 5th toes, and VSD were performed. After 3 weeks, there was a large soft-tissue defect of the soles and arches, with the 3rd to 5th toes missing. Given the extensive nature of the defect, we used flow-through technology to connect the MPAPF in series with the ALTPF. The MPAPF was used to repair the anterior plantar defect, whereas the ALTPF was used to repair the remaining plantar defect. The vascular pedicle of the ALTPF was then anastomosed with the posterior tibial artery and its accompanying vein. Free skin grafting was performed for a few small wounds on the dorsum of the foot. All the wounds were repaired in a single stage, and the flaps survived well. Postoperative follow-up at 14 months revealed that the flaps had good color and survival and that sensation had recovered (Figure 2).

## Discussion

The foot is the body’s load-bearing and locomotive organ; its plantar skin is particularly thick and tough and provides good tactile sensation. The thickness of subcutaneous connective tissue in the foot serves as an effective cushioning system. The dense interstitial fibrous tissue closely connects the skin with the calcaneus and the deep fascia of the sole, maintaining excellent stability of the skin. These unique structural characteristics enable it to perform various functions, such as load-bearing, walking, and shock absorption.^18^ The sole of the foot can be divided into load-bearing and nonload-bearing areas according to its function.^19^ Soft-tissue defects in different areas need to be repaired according to their respective characteristics to achieve satisfactory appearance and function. Soft-tissue defects in the nonload-bearing area can be covered with skin grafts. In contrast, the repair requirements for defects in the load-bearing area are greater, including the following: (1) the repaired area needs to have a relatively normal appearance and allow the patient to wear ordinary shoes; (2) the tissue should have a certain thickness, hardness, and durability and provide a protective sensation that can resist pressure and wear; (3) it should have a good anchoring function with deep tissues and be able to resist shear forces and meet the demands of walking^20^; and (4) it needs to have a rich blood supply, be less prone to frostbite, and be conducive to wound healing. Therefore, the management of complex forefoot defects demands a reconstructive solution that addresses the dual challenges of durable, sensitive coverage and the preservation of gait mechanics. In this clinical series, we explored the feasibility of a combined free MPAPF and ALTPF approach for the treatment of these formidable injuries. Our preliminary findings indicate that this dual-flap strategy could successfully achieve stable wound closure, facilitate early functional recovery, and restore protective sensation without major complications or flap loss in our small cohort.

The primary finding of this study was the successful restoration of protective sensation in all patients, with the majority (83.3%) achieving it within 6 months. These outcomes underscore the critical advantage of employing innervated flaps such as the MPAPF in weight-bearing reconstruction. Medial plantar nerve coaptation likely plays a pivotal role in this process by facilitating the return of protective sensibility, which is fundamental for preventing neuropathic ulceration.

Furthermore, using similar tissues to repair soft-tissue defects is important. For the sole, there is no tissue with similar properties in any other part of the body, except the palm and the sole itself. Therefore, using tissue from the sole is the ideal choice for repairing sole defects. Owing to its unique biomechanical properties, including abrasion resistance, shock absorption, and sensory restoration, the medial plantar flap is a superior option for addressing the challenges posed by forefoot reconstruction.^21^ This flap offers structural and functional compatibility with plantar tissues, making it highly effective for weight-bearing applications. The thick, glabrous skin of the medial plantar flap provides exceptional resistance to friction and pressure, which is essential for maintaining durability in areas exposed to high mechanical stress. Additionally, the inclusion of shock-absorbing fibrofatty tissue and fibrous septa ensures effective load distribution, closely mimicking the natural plantar architecture and minimizing the risk of complications such as ulceration and flap breakdown,^22^ which is likely responsible for the observed long-term durability and absence of ulceration in our patients during the follow-up period.

Compared with other reconstruction techniques, the MPAPF has clear advantages in terms of durability and functionality. Although they are commonly used, reverse sural artery flaps are prone to vascular crisis because of their large size, which often leads to necrosis. Additionally, their inability to restore sensation increases the risk of ulceration, making them unsuitable for load-bearing areas.^23^, ^24^, ^25^ Free flaps, while often employed for large defects requiring volume replacement, lack the durability and sensory recovery required for plantar reconstruction.^26^ Skin grafts, although straightforward for superficial defects, are generally inadequate for plantar reconstruction because of their lack of shock-absorbing properties and susceptibility to mechanical stress, which can lead to thinning and eventual failure.^27^ We believe that the advantages of using the MPAPF for forefoot reconstruction in our study were as follows: (1) The medial plantar flap was harvested from a nonload-bearing and hidden area. After flap harvesting, the donor site can be closed with a skin graft, which has a minimal impact on the load-bearing function and appearance of the foot. (2) The skin thickness and tissue structure of the MPAPF are similar to those of the forefoot load-bearing area, providing wear and pressure resistance that meet the load-bearing requirements of the forefoot. (3) The vascular and nerve anatomy of the flap are consistent, allowing the proximal end of the flap to be used as a pedicle for repairing heel defects and the distal end to be used as a pedicle for repairing forefoot defects.

The MPAPF offers unparalleled advantages in repairing small soft-tissue defects of the forefoot or hindfoot. However, it also has several limitations, such as a small flap size and limited coverage area, which often restrict its use as a solitary flap for extensive defects. Previous reports have focused primarily on the repair of small-area hand injuries,^28^, ^29^ with very few reports on complex large-area reconstruction of the foot. In our case series, the defect area of the foot was large, and the MPAPF alone could not completely cover the defect. Therefore, we combined an ALTPF with an MPAPF to address these limitations. In this approach, the MPAPF provides durable coverage for the weight-bearing zone, whereas the ALTPF contributes additional bulk for extended coverage. This combination achieved satisfactory results. Furthermore, the low donor-site morbidity associated with the MPAPF enhances its utility. The nonweight-bearing instep area allows for straightforward donor-site closure with minimal functional or aesthetic impact, a notable advantage over techniques such as sural artery flaps, which can leave visible scars and functional limitations, but harvesting extensive flaps may increase the risk of donor site complications. Potential issues include delayed wound healing, skin graft loss, hypertrophic scarring, and, in rare cases, functional deficits or persistent pain that could affect ambulation. In our series, all donor sites healed without major complications, this may be related to the small number of cases in our series; however, we acknowledge that careful patient selection, meticulous surgical technique, and comprehensive postoperative care are essential to minimize donor site morbidity.

Although we achieved satisfactory clinical outcomes, this study has notable limitations. Given that this was a retrospective case series with a small sample size (*n* = 6) and no control group, our ability to validate the superiority of this technique is limited. Although the results are promising, they remain preliminary. Additionally, some ALTPFs may require debulking because of excessive bulkiness, thereby increasing the risk of secondary surgery for patients. Furthermore, amputation may be a reasonable alternative in select cases, particularly when patient comorbidities, functional expectations, or resource constraints favor a simpler solution. However, the decision between limb salvage and amputation must be individualized, taking into comprehensive consideration patient factors, cultural preferences, injury patterns, and surgical resources. In our cohort, all patients demonstrated strong motivation for limb salvage, and our technique provided a viable pathway; however, this approach may not be universally applicable.

Future directions should include prospective, comparative studies with larger cohorts and long-term follow-up to objectively evaluate functional and sensory outcomes compared with those of other established methods. The integration of preoperative vascular mapping and the use of neurotrophic agents to accelerate sensory recovery could further enhance the efficacy of this procedure. Additionally, innovations in tissue engineering and flap augmentation may expand its application to more complex and extensive defects. Comparative multicenter studies focusing on long-term durability, sensory recovery, and functional outcomes are essential to refine surgical protocols and establish clearer guidelines for flap selection in plantar reconstruction. Furthermore, technical refinements, such as optimizing microsurgical anastomosis and enhancing venous drainage, can address potential complications such as venous congestion and partial flap necrosis, ensuring consistently favorable outcomes.

## Conclusion

The combination of the MPAPF and ALTPF is a technically feasible and promising strategy for complex forefoot reconstruction, providing durable coverage and sensory restoration. Although these preliminary results are encouraging, further validation through larger comparative studies is warranted to definitively establish the efficacy of this procedure.

## Abbreviations

MPAPF, medial plantar artery perforator flap; ALTPF, anterolateral thigh perforator flap; VSD, vacuum sealing drainage; DPA, dorsal pedis artery; PTA, posterior tibial artery.

## Funding

The National Natural Science Foundation of China (82372381) and the Basic Research Joint Project of Kunming Medical University (202301AY070001-051) provided funding for this study.

## Availability of data and materials

The database used during the current study is available from the corresponding author upon reasonable request.

## Authors’ contributions

Conception and design: KXD, XQH, and YLZ; collection and assembly of the data: DHZ and FYJ; data analysis and interpretation: YJL and WLH; surgical instructions: DHZ and YLZ; manuscript writing: YLZ and KXD; manuscript revision: KXD, YLZ, and DHZ; and final approval of the manuscript: all the authors.

## Ethics approval and consent to participate

This study was a single-center consecutive case series analysis approved by the ethics committee of the 920th Hospital of the Joint Logistics Support Force of the Chinese PLA. The study protocol was performed in accordance with the Declaration of Helsinki (as revised in 2013).

## Consent for publication

All the patients whose case reports are presented in this manuscript provided consent for publication.

## Declaration of competing interests

The authors declare that they have no competing interests.

## References

[1] Gu J.X., Huan A.S., Zhang N. (2017). Reconstruction of heel soft tissue defects using medial plantar artery island pedicle flap: clinical experience and outcomes analysis. J Foot Ankle Surg.

[2] Zgonis T., Cromack D.T., Stapleton J.J. (2007). Utilizing a crossover reverse sural artery flap for soft tissue reconstruction of the plantarforefoot after a severe degloving injury. Int J Low Extrem Wounds.

[3] Leclère F.M., Casoli V. (2015). Reconstruction of a traumatic plantar foot defect with a novel free flap: the medial triceps brachii free flap. J Cosmet Laser Ther.

[4] Zhou J.D., Zhang X.F., Zhang Y.X., Wang W.C., Xu Y.J. (2022). Reconstruction of complex plantar forefoot defects using free tissue flaps combined with contralateral instep thick skin grafts. J Orthop Surg (Hong Kong).

[5] Fu D.H., Zhou L.Y., Yang S.H., Xiao B.J. (2013). Surgical technique: repair of forefoot skin and soft tissue defects using a lateral tarsal flapwith a reverse dorsalis pedis artery pedicle: a retrospective study of 11 patients. Clin Orthop Relat Res.

[6] Tuluy Y., Özkaya Ünsal M., Bali U., Parspancı A., Ünal D. (2024). Reconstruction of plantar foot defects with free super-thin anterolateral thigh flap. ANZ J Surg.

[7] Sayyed A.A., Towfighi P., Deldar R., Attinger C.E., Evans K.K. (2022). Free flap reconstruction of plantar weight-bearing heel defects: long-term functional and patient-reported outcomes. Microsurgery.

[8] Harrison D.H., Morgan B.D. (1981). The instep island flap to resurface plantar defects. Br J Plast Surg.

[9] Koshima I., Narushima M., Mihara M. (2007). Island medial plantar artery perforator flap for reconstruction of plantar defects. Ann Plast Surg.

[10] Lai C.H., Lai C.S., Huang S.H., Lin S.D., Chang K.P. (2010). Free medial plantar artery perforator flaps for the resurfacing of thumb defects. Ann Plast Surg.

[11] Zelken J.A., Lin C.H. (2016). An algorithm for forefoot reconstruction with the innervated free medial plantar flap. Ann Plast Surg.

[12] Lohasammakul S., Turbpaiboon C., Chaiyasate K. (2018). Anatomy of medial plantar superficial branch artery perforators: facilitation of medial plantar superficial branch artery perforator (MPAP) flap harvesting and design for finger pulp reconstruction. Microsurgery.

[13] Lofstrand J.G., Lin C.H. (2018). Reconstruction of defects in the weight-bearing plantar area using the innervated free medial plantar (instep) flap. Ann Plast Surg.

[14] Scaglioni M.F., Rittirsch D., Giovanoli P. (2018). Reconstruction of the heel, middle foot sole, and plantar forefoot with the medial plantar artery perforator flap: clinical experience with 28 cases. Plast Reconstr Surg.

[15] Wong C.H., Ong Y.S., Wei F.C. (2012). The anterolateral thigh - vastus lateralis conjoint flap for complex defects of the lower limb. J Plast Reconstr Aesthet Surg.

[16] Saint-Cyr M., Schaverien M., Wong C. (2009). The extended anterolateral thigh flap: anatomical basis and clinical experience. Plast Reconstr Surg.

[17] Shi Y., Xu Y., Zhu Y. (2022). Microsurgical anterolateral thigh flap for reconstruction of extremity soft tissue defects in pediatric patients. Ann Plast Surg.

[18] Heymans O., Verhelle N., Lahaye T. (2005). Covering small defects on the weight bearing surfaces of the foot: the free temporal fasciocutaneous flap. Br J Plast Surg.

[19] May J.W., Halls M.J., Simon S.R. (1985). Free microvascular muscle flap with skin graft reconstruction of extensive defects of the foot: a clinical and gait analysis study. Plast Reconst Surg.

[20] Sinha A.K., Wood M.B., Irons G.B. (1989). Free tissue transfer for reconstruction of the weight-bearing portion of the foot. Clin Orthop Relat Res.

[21] Siddiqi M.A., Hafeez K., Cheema T.A., Rashid H.U. (2012). The medial plantar artery flap: a series of cases over 14 years. J Foot Ankle Surg.

[22] Oh S.J., Moon M., Cha J., Koh S.H., Chung C.H. (2011). Weight-bearing plantar reconstruction using versatile medial plantar sensate flap. J Plast Reconstr Aesthet Surg.

[23] Mahmoud W.H. (2017). Foot and ankle reconstruction using the distally based sural artery flap versus the medial plantar flap: a comparative study. J Foot Ankle Surg.

[24] Park J.H., Choi I.C., Hong T.C., Kang J.W., Park J.W. (2021). Reconstruction of the weight-bearing heel with nonsensate reverse sural artery flaps. Injury.

[25] Nangole F.W., Ogallo J.P., Muoke A. (2021). Reliability of reverse sural flap in the reconstruction of midfoot and forefoot defects. J Foot Ankle Surg.

[26] Jiga L.P., Jandali Z., Merwart B., Skibinska K. (2020). The free vastus lateralis muscle flap. A smart less used flap for soft tissue reconstruction of the weight-bearing foot. Injury.

[27] Khan F.H., Beg M.S.A., Obaid-Ur-Rahman (2018). Medial plantar artery perforator flap: experience with soft-tissue coverage of heel. Plast Reconstr Surg Glob Open.

[28] Rodriguez-Vegas M. (2014). Medialis pedis flap in the reconstruction of palmar skin defects of the digits: clarifying the anatomy of the medial plantar artery. Ann Plast Surg.

[29] Xu X., Wang C., Chen Z., Li J. (2022). Medial plantar artery perforator (MPAP) flap is an ideal option for reconstruction of complex soft tissue defect in the finger: clinical experience from 11 cases. Front Surg.

